# How Sustained is Roux-en-Y Gastric Bypass Long-term Efficacy? Roux-en-Y Gastric Bypass efficacy

**DOI:** 10.1007/s11695-021-05458-y

**Published:** 2021-05-22

**Authors:** Marta Guimarães, Catarina Osório, Diogo Silva, Rui F. Almeida, António Reis, Samuel Cardoso, Sofia S. Pereira, Mariana P. Monteiro, Mário Nora

**Affiliations:** 1 Endocrine, Cardiovascular & Metabolic Research, Unit for Multidisciplinary Research in Biomedicine (UMIB), University of Porto, Porto, Portugal; 2 Department of Anatomy, Institute of Biomedical Sciences Abel Salazar (ICBAS), University of Porto, Jorge Viterbo Ferreira 228, Building 1.3, 4050-313 Porto, Portugal; 3 Department of General Surgery, Centro Hospitalar de Entre o Douro e Vouga, Cândido Pinho, Santa Maria da Feira, Portugal

**Keywords:** Roux-en-y gastric bypass, Obesity, Type 2 diabetes

## Abstract

**Purpose:**

The rate of weight regain after Roux-en-Y Gastric Bypass (RYGB) can hamper the procedure long-term efficacy for obesity treatment and related comorbidities. To evaluate the rate of weight loss and comorbidity remission failure 10 years or more after RYGB surgery.

**Materials and methods:**

Retrospective observational cohort study. Patients submitted to RYGB for obesity treatment at a single centre with 10 years or more after surgery underwent a clinical reassessment.

**Results:**

Among the subjects invited for clinical revaluation (*n* = 585), only those who performed RYGB and attended the hospital visit were included in the study (*n* = 281). The pre-operative mean body mass index (BMI) was 44.4 ± 6.1 kg/m^2^. Mean post-operative time was 12.2 ± 1.1 years. After surgery, mean BMI was significantly lower 33.4 ± 5.8 kg/m^2^ (*p* < 0.0001), 29.5% with a BMI < 30 kg/m^2^. Mean Total Weight Lost (%TWL) was 24.3 ± 11.4%, reaching a %TWL ≥ 20% in 70.1% with a mean %TWL of 30.0 ± 7.0%. Co-morbidities remission rate was 54.2% for type 2 diabetes, 34.1% for hypertension, 52.4% for hyperlipidemia and 50% for obstructive sleep apnea. Early complications rate was 13.2% and revision surgery occurred in 2.8% of patients. Four patients died of RYGB complications within the first 90 days after surgery.

**Conclusion:**

RYGB has a high rate of long-term successful weight loss and obesity-associated comorbidity improvement. Weight loss failure requiring revision surgery occurs in a small proportion of patients. Our data confirms the long-term effectiveness of RYGB as primary bariatric intervention.

**Graphical abstract:**

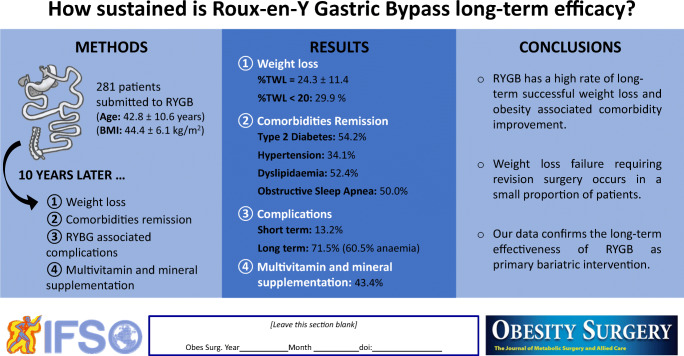

## Introduction

A bariatric surgeon can choose between different procedures that are now available for the treatment of patients with obesity and related co-morbid conditions [1].

The gradient of surgical technical complexity, weight-loss effectiveness, co-morbidity improvement and risk surgery-derived complications has been described as follows: sleeve gastrectomy (SG), Roux-en-Y Gastric Bypass (RYGB), single anastomosis duodenal ileal bypass with sleeve gastrectomy (SADI-S) and biliopancreatic diversion/duodenal switch (BPD/DS), from the lowest to the highest [2–4]. The favourable benefit-to-risk ratio of RYGB, given the long-term excess weight loss achieved and obesity-related co-morbidity improvement, as well as the relatively low risk of complications when compared to other bariatric interventions, led some authors to recommend RYGB as the gold standard bariatric surgery procedure [5, 6].

In order to be considered a gold standard technique, a bariatric procedure must prove to be effective in achieving and maintaining clinically relevant weight loss over the long term, as well as inducing and sustaining the remission or significant improvement of obesity associated co-morbidities along with a low morbidity and mortality rate attributed to short and long-term surgery-derived complications, and these include the risk of micronutrient deficiencies. Ideally, a bariatric surgery procedure should have a minimum interference with micronutrient absorption, in order to avoid complications derived from erratic absorption and uncertain compliance with routine vitamin supplementation. Additionally, the bariatric surgery procedure should be technically feasible, being easy to adapt and modify according to individual anatomical features [7].

According to the 4^rd^ IFSO Global Registry Report, between 2014 and 2018, the most frequent procedure performed worldwide was SG, representing 46% of bariatric surgeries and followed by RYGB in 38% of the interventions [8]. As a matter of fact, the proportion of RYGB procedures performed worldwide has been gradually decreasing over the past decade, along with an inexorable rise in the rate SG performed globally.

RYGB was first described in 1967 by Mason [9]. Early reports with long-term patient outcomes after RYGB, consistently demonstrated that the weight loss achieved was significant and sustained, along with a high rate of obesity related co-morbidities improvement or even clinical remission [10–15].

However, more recent reports on weight regain rates occurring more frequently 3 years after RYGB has raised doubts over the previously established long-term efficacy of the procedure [16]. The technical difficulty of converting RYGB procedure into another bariatric intervention is also appointed as one of the major limitations of this bariatric intervention. Moreover, revision bariatric surgery is technically complex, has a high morbidity rate and lower efficacy when compared to primary bariatric interventions [17, 18].

Reports on long-term complications after RYGB, such as chronic or recurrent abdominal pain, dumping syndrome and post-bariatric hypoglycemia, driving the need of hospital re-admission or surgical re-interventions in an estimated rate of 21.9% within 4 years after surgery [19], contributed for raising the controversy on the procedure safety.

Given the notorious change in preference of bariatric surgery procedure performed worldwide, our goal was to conduct a clinical revaluation of patients 10 years or more after being submitted to RYGB for obesity treatment. Our primary aim was to assess the rates of weight loss and comorbidity remission failure that could point towards the need to change the current preference for RYGB in detriment of other interventions, while trying to identify patient characteristics associated with poorer weight loss over the long term and eventual need for revision surgery.

## Methods

### Patients’ and methods

**Fig. 1:**
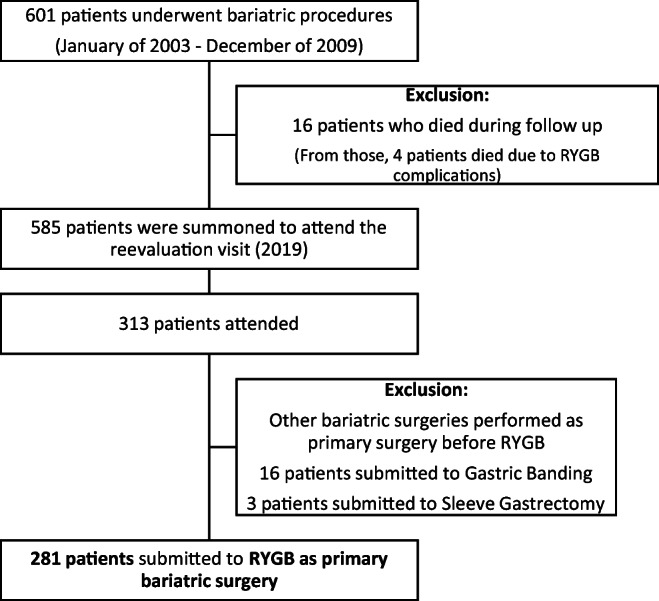
Patient selection flowchart

This was a retrospective observational cohort study. Data concerning patients (*n* = 601) submitted to bariatric surgery for obesity treatment with a body mass index (BMI) > 35 kg/m^2^ with or without comorbidities, between January of 2003 and December of 2009, in a single centre based at a public hospital, who completed 10 years or more after surgery, was retrieved for analysis from our bariatric patients cohort. After excluding patients that died during follow-up time (*n* = 16) from the total number of patients with that underwent bariatric surgery, the remaining patients (*n* = 585) were invited to attend an outpatient in-hospital visit during the year of 2019. Clinical reassessment included a full medical history, physical examination and fasting blood collection for laboratory analysis. Patients that refused to come for an in-person office visit were excluded from the study. Among the eligible patients that attended the clinic (*n* = 313), only those submitted to RYGB surgery as primary intervention (*n* = 281) were considered for statistical analysis (Fig. 1).

This study was approved by the institutional ethical review board and all patients signed an informed consent document before conducting any study assessment and enrolment.

### Surgical technique

The surgical procedure consisted of performing a standard RYGB with a variable biliopancreatic limb length (50–200) and a constant 120 cm alimentary limb length as previous reported [20].

### Routine post-operative follow-up

After bariatric surgery, all patients were instructed to maintain lifelong multivitamin and vitamin D supplements. Post-operative follow-up consisted of multidisciplinary office visits until the 3rd year after surgery, followed by management by the general practitioner thereafter. Standard post-RYGB supplementation protocol consisted of multivitamin/mineral preparation chewable tablets according to the ASMBS Guidelines recommendations [21], for lifelong and vitamin D doses adapted to individual patient needs.

### Revaluation follow-up visit

Follow-up time was calculated for each patient as the time elapsed since surgery until revaluation appointment 10 years or more after intervention.

The parameters evaluated during hospital visit were body weight, BMI, percentage of Excess Weight Lost (%EWL), percentage of excess BMI lost (%EBMIL), percentage of Total Weight Lost (%TWL), early surgical complications (first 90 days after surgery), long term surgical complications (over 90 days after surgery), presence of obesity-related comorbidities and ongoing treatment, cancer diagnosis after surgery and history of revision bariatric surgery performed during the follow-up period.

Fasting blood test for post-bariatric patients follow-up, included full blood count, fasting blood glucose, glycated haemoglobin (HbA1c), albumin, total proteins, iron kinetics, folate, lipid profile, micronutrients levels (vitamin A, B1, B6, B9, B12 and D), intact parathyroid hormone and calcium.

The patients were classified as being in remission of previous comorbid conditions if diagnostic criteria for the disease were no longer present while off any specific drugs. Patients were considered as having relapse of previous comorbid conditions when diagnostic criteria of the disease were found to be present after a period of remission.

### Outcomes

The primary surgical outcome was weight loss, quantified using percentage total weight loss (%TWL), percentage excess weight loss (%EWL), and percentage Excess BMI Loss (%EBMIL), identifying patients who failed to achieve this goal 10 years or more after intervention, or who needed revision surgery for weight loss failure. The rate of patients with unsuccessful weight loss at 10 years or more was calculated using a %TWL<20.

Secondary surgical outcomes included obesity related comorbidity status, RYGB-associated morbidity and mortality, and adherence to multivitamin supplementation. All complications identified were included in the analysis.

### Statistical analysis

Categorical variables are expressed as number of cases and percentage (%), and the quantitative variables are expressed as mean and standard deviation. X^2^ test was used for the analysis of categorical variables. For quantitative variables, the difference between 2 independent experimental groups was evaluated using the unpaired Student *t* test for normally distributed variables, and the Mann–Whitney *U* test for variables that did not meet the normal parameters.

Linear regression was used to identify independent associations between pre-operative variables and the % TWL at follow-up. Binary logistic regression was used in multivariate analysis to identify pre-operative predictors of long-term unsuccessful weight loss.

A *p* value <0.05 was considered statistically significant. All statistical analyses were performed with the aid of the GraphPad Prism software version 8.0 and IBM SPSS Statistics version 24, both for Windows.

## Results

This study describes the long-term outcomes of a cohort of patients submitted to RYGB (*n* = 281), via laparotomy (*n* = 4) or laparoscopy (*n* = 277), at a single centre. Among these patients, 241 (85.8%) were females and 40 (14.2%) were males, with a mean age of 42.8 ± 10.6 years, ranging between 20 and 67 years, at the time of surgery. The pre-operative mean BMI was 44.4 ± 6.1 kg/m^2^ with 44% (*n* = 124) of patients with BMI > 45 kg/m^2^ and 14.6% (*n* = 41) with BMI > 50 kg/m^2^.

The average follow-up time after surgery was 12.2 ± 1.1 years. After surgery BMI decreased significantly to 33.4 ± 5.8 kg/m^2^ (*p* < 0.0001), with 29.5% (*n* = 83) of the patients reaching a BMI < 30 kg/m^2^. Overall, 64.8% of the patients achieved a BMI < 35 kg/m^2^, 10 years or more after RYGB. Mean total weight lost (%TWL) was 24.3 ± 11.4, with 70.1% (*n* = 182) of patients reaching %TWL ≥ 20% with a mean %TWL of 30.0 ± 7.0% (Table 1). Co-morbidities remission rate 10 years or more after RYGB were as follows: 54.2% for T2D, 34.1% for hypertension, 52.4% for dyslipidemia and 50% for OSA. Additionally, metabolic profile was significantly improved with decreased fasting glucose, total cholesterol, LDL cholesterol and triglycerides, and increased HDL cholesterol (Table 1).

**Table 1:** Demographic data. Analytical profiles of patients who underwent RYGB before surgery and at 10 or more years of follow-up. Rates of comorbidities remission, relapse, and new diagnosis

Variable	Pre-operative	10 or more years	*p value*
N	281		*-*
Average follow up time *(years)*	12.2 ± 1.1		*-*
Sex *(M:F)*	40:241 (85.8% female)		*-*
Age *(years)*	42.8 ± 10.6	55.5 ± 10.7	*p<0.0001*
Weight (kg)	115.0 ± 16.7	86.6 ± 16.2	*p<0.0001*
BMI (kg/m^2^)	44.4 ± 6.1	33.4 ± 5.8	*p<0.0001*
BMI ≤ 35	8 (2.8%)	182 (64.8%)	
35 < IMC ≤ 40	62 (22.1%)	65 (23.1%)	
40 < IMC ≤ 45 45 < IMC ≤ 50 IMC > 50	87 (31.0%) 83 (29.5%) 41 (14.6%)	22 (7.8%) 10 (3.6%) 2 (0.7%)	*p<0.0001*
EBMIL (%)	-	57.1 ± 30.0	*-*
TWL (%)	-	24.3 ± 11.4	*-*
EWL (%)	-	57.1 ± 30.0	*-*
Haemoglobin (g/dL)	13.6 ± 1.1 (n = 241)	12.4 ± 1.5 (n = 255)	*p<0.0001*
Iron (ug/dL)	74.8 ± 28.5 (n = 77)	75.2 ± 37.8 (n = 209)	*p = 0.7885*
Fasting glucose (mg/dL)	109.6 ± 37.8 (n = 243)	95.4 ± 31.3 (n = 238)	*p<0.0001*
Lipid profile			
Total Cholesterol HDL Cholesterol LDL Cholesterol Triglycerides	199.4 ± 34.3 (n = 217) 45.8 ± 10.1 (n = 212) 127.0 ± 27.7 (n = 211) 133.5 ± 73.1 (n = 214)	185.7 ± 30.2 (n = 238) 61.3 ± 14.2 (n = 238) 104.9 ± 26.4 (n = 238) 97.5 ± 45.9 (n = 238)	*p<0.0001* *p<0.0001* *p<0.0001* *p<0.0001*
Type 2 diabetes Remission Relapse New Diagnosis	83 (29.5%) - - -	44 (15.7%) 45 (54.2%) 17 (20.5%) 6 (3.0%)	*p < 0.0001*
Hypertension Remission Relapse New Diagnosis	129 (45.9%) - - -	103 (36.7%) 44 (34.1%) 16 (12.4%) 18 (11.8%)	*p = 0.0321*
Dyslipidaemia Remission Relapse New Diagnosis	84 (29.9%) - - -	50 (17.8%) 44 (52.4%) 8 (9.5%) 10 (5.1%)	*p = 0.0010*
OSA Remission Relapse New Diagnosis	44 (15.7%) - - -	23 (8.2%) 22 (50.0%) 0 (0.0%) 1 (0.4%)	*p = 0.0088*

Early complications occurred in 13.2% (*n* = 37) of patients, 6.5% classified as Clavien-Dindo I or II. The most prevalent complications were gastrojejunal anastomosis related (*n* = 14) and surgical wound infection (*n* = 10). Reoperation within the first 90 days after surgery was needed in 8 patients (2.8%) (Table 2).

**Table 2:** Early and Long-term complications in patients after RYGB

Short term complications	
Number of patients with short term complications (<90 days) Surgical wound infection Gastrojejunal anastomosis fistula Gastrojejunal anastomosis stenosis Gastrojejunal anastomosis leak Intraabdominal abscess Respiratory failure Food intolerance Hemoperitoneum Pulmonary embolism Jejunal perforation Peritonitis Digestive bleeding	37 (13.2%) 10 (3.5%) 8 (2.7%) 3 (1.1%) 3 (1.1%) 3 (1.1%) 2 (0.7%) 2 (0.7%) 2 (0.7%) 1 (0.4%) 1 (0.4%) 1 (0.4%) 1 (0.4%)
Clavien-Dindo Classification	
Grade I Grade II Grade IIIa Grade IIIb Grave IVa Grade IVb	11 (4.0%) 7 (2.5%) 6 (2.1%) 9 (3.2%) 4 (1.4%) 0 (0.0%)
Number of reoperations at 90 days	8 (2.8%)
Long term complications	
Number of patients with long term complications Iron deficiency anaemia Oral iron therapy Intravenous iron therapy Blood transfusion therapy Anterior abdominal wall hernia Hypoglycaemia Bowel obstruction (adhesions) Gastrojejunal anastomosis stenosis Internal hernia Perforated hollow viscus Chronic diarrhoea Vitamin deficiencies	201 (71.5%) 170 (60.5%) 27 (33.8%) * 42 (52.4%) * 11 (13.8%) * 24 (8.5%) 13 (4.2%) 9 (3.2%) 7 (2.5%) 4 (1.4%) 3 (1.1%) 5 (1.8%) 1 (0.4%)
Intestinal malabsorption syndrome (Hypoproteinaemia and	
Hypoalbuminaemia) Pathological fractures Pulmonary embolism	1 (0.4%) 1 (0.4%) 1 (0.4%)
Number of patients with two or more long term complications	38 (13.5%)

(*This values refer to percentage of patients with anaemia secondary to iron deficiency that required each of the following treatment interventions: oral iron, intravenous iron or blood transfusion)

Long-term complications were reported in 201 patients (71.5%). Iron deficiency, the most frequent long-term complication occurred in 170 patients (60.5%), of which only 80 patients (47.1%) were under mineral/multivitamin supplementation after surgery. At the time of the revaluation visit, only 43.4% (*n* = 122) of the patients were under mineral or multivitamin supplementation.

Anterior abdominal wall hernias (*n* = 24) and post-bariatric hypoglycaemia (*n* = 13) were the other most common long-term complications observed. Two or more long-term complications occurred in 38 patients (Table 2).

After the primary bariatric intervention, 2.8% (*n* = 8) of the patients needed revision surgery for secondary weight loss failure. None of the revision surgery interventions was performed due to excessive weight loss or severe malnutrition, intractable dumping syndrome or chronic abdominal pain.

To identify the factors associated with weight loss failure, defined as not reaching the TWL ≥ 20%, a univariate and multivariate analysis comparing the patients with a successful versus non-successful weight loss, including age, initial BMI, T2D, hypertension, dyslipidemia, OSA, depression, smoking and laboratory parameters comprising glucose, hemoglobin A1c, total and HDL cholesterol, triglycerides, insulin and C-Peptide. None of the evaluated parameters was found to be associated with higher probability of weight loss failure.

From the initial cohort of 601 patients, 4 patients submitted to RYGB, died within the first 90 days after surgery: 3 patients from septic shock after gastrojejunal anastomosis leak and 1 patient from acute kidney injury.

## Discussion

The primary aim of this study was to evaluate the rates of efficacy and failure 10 years or more after RYGB surgery, when performed as primary bariatric intervention for the treatment of obesity and obesity related diseases.

To define a successful bariatric surgery intervention several factors should be considered, including primarily long term weight loss, but also obesity comorbidities improvement, impact on quality-of-life and surgical associated morbidity. However, there is no universal consensus on how to define successful surgical induced weight loss. The majority of bariatric surgery efficacy criteria refer to short and medium term weight loss, which as being static are not necessarily appropriate for patients with longer follow-up times. Maximum weight loss is observed at 1 to 2 years after surgery with a small weight regain occurring after this initial postsurgical period [22]. Additionally, patients with higher pre-operative BMIs are also less likely to reach a normal weight [23]. Among the existing criteria, EWL ≥ 50%, BMI reduction > 10 kg/m^2^, Alterable Weight Loss (AWL) ≥ 35%, and TWL ≥ 20% or ≥25% stand out as most commonly used [24].

Weight loss failure, defined as losing less than 20% of the total weight at 10 or more years after the procedure occurred in 29.9% of the patients. Similar results were observed by other authors, in cohorts with similar follow-up periods [16, 25].

Ten years or more after surgery, revision surgery due to secondary weight loss failure only occurred in 2.8%. We also sought to identify baseline clinical or laboratory predictors of insufficient weight loss outcome in the proportion of patients that did not reach the TWL ≥ 20% goal. However, no failure predictors were identified.

After surgery, the prevalence of obesity related comorbidities was also significantly lower, highlighting the impact of RYGB on this population overall health. The operation not only provided durable weight loss but also long-term comorbidity amelioration, in particular T2D remission observed in 54.2% of this patient cohort. In our previous patient series report of 94 patients with obesity and T2D with preserved pancreatic function, namely with a relatively short T2D disease duration before surgery, reasonable pre-operative glycemic control as assessed by HbA1C levels and small proportion of patients requiring insulin therapy for disease control, RYGB with 200 cm biliopancreatic limb resulted in a diabetes remission rate of 87.91% at 6 months, 92.68% at 12 months, 92.85% at 24 months, and 100% at 36 months of follow-up [26]. The current study includes 45 patients of the previous report with a longer follow-up time besides other patients with T2D that were submitted to RYGB with shorter biliopancreatic limb length, thus the overall lower diabetes remission rate observed.

In the early post-operative period, minor complications were more common, while major morbidity and mortality were low and comparable with other major published data [27, 28]. Surgical mortality occurred predominantly in the beginning of our centre learning surgical curve. The most prevalent long-term complications, were incisional hernias and iron deficiency. Incisional hernias occurred in a proportion that is perfectly matched with other series published, although in our series, these were more frequent in patients submitted to laparotomy that had early infectious complications [29].

In our series, we report a 60.5%, prevalence of iron deficiency after RYGB, which is higher than the 18 to 53% prevalence reported in the literature [30]. After RYGB, iron deficiency can be attributed to several risk factors including diminished gastric acid secretion combined with exclusion of the duodenum from the alimentary tract after gastric bypass surgery, red meat food intolerance, and increased blood losses in premenopausal women [31, 32]. Additionally, 30 to 40% of the patients were demonstrated to have micronutrient deficiencies including low serum iron concentrations prior to bariatric surgery, which may be aggravated and further contribute for anemia after surgery [33]. After RYGB, patients are routinely advised to maintain lifelong multivitamin supplements to compensate for the reduced absorption and prevent the occurrence of deficiencies. Furthermore, patients and general practitioners are instructed to monitor micronutrient blood levels routinely on a yearly basis. Despite these standard practice recommendations, only 43.4% of the patients were found to remain adherent to multivitamin/mineral supplementation 10 years or more after RYGB. Previous studies also report a sub-optimal rate of multivitamin adherence, varying between 64 and 92% at short term, and falling significantly to 33% over a 10-year period [34–36]. Possible reasons for the low long-term adherence to multivitamin supplementation include the lifelong economic costs along with the lack of perceived benefit, once micronutrient deficiencies do not have consequences that are immediately recognizable by the patients.

In summary, our study shows that RYGB has a high rate of long-term successful weight loss and obesity associated comorbidity improvement, reinforcing previous similar reports [11, 12, 15, 37–42]. The long-term morbidity was overall low, despite there is a possibility of complications that may affect quality of life, such as dumping syndrome, internal hernias, marginal ulcers or severe vitamin deficiencies, which have been appointed as reasons for progressive abandonment of the RYGB. Weight loss failure requiring revision surgery occurred only in a small proportion of patients.

Although this study includes a large number of patients with a long-term follow-up, we acknowledge that it presents some limitations that must be acknowledged, these include the risk of bias inherent to an observational retrospective study and the fact that some patients who previously submitted to RYGB did not accept the invitation sent by post to attend the reevaluation appointment 10 years after surgery and so were not included in the data analysis and interpretation.

## Conclusion

RYBG has a high rate of long-term successful weight loss and obesity associated comorbidity improvement, while surgical related morbidity is low and weight loss failure requiring revision surgery occurs only in a small proportion of patients. Therefore, the available data does not provide evidence to support the reasons appointed for displacing RYGB procedure as a first line primary intervention for obesity treatment.
